# Match for Orthopedic Fellowship Programs in the United States: Online Accessibility, Content, and Accreditation Comparison Between Subspecialties and Review of Alternative Resources

**DOI:** 10.7759/cureus.19643

**Published:** 2021-11-16

**Authors:** Mohit J Jain, Karthikeyan Chinnakkannu, Dhavalkumar J Patel, Sivashanmugam Raju

**Affiliations:** 1 Orthopedic Sports Medicine, Jefferson 3B Orthopedics, Philadelphia, USA; 2 Orthopedic Sports Medicine, Plancher Orthopaedics & Sports Medicine, New York, USA; 3 Reconstructive Orthopedic Surgery, Laredo Physicians Group, Laredo, USA; 4 Department of Orthopedics, Saint Louis University School of Medicine, St. Louis, USA

**Keywords:** accessibility, accreditation, content, online, orthopedic fellowship, sponsoring society, subspecialty, acgme, freida, sf match

## Abstract

Background: Orthopedic surgery is considered among the highly competitive medical specialties to get in as a career in the United States. San Francisco Match (SF Match) is the matching service for orthopedic subspecialty fellowship programs, and the internet is the main source for applicants to obtain program information in the modern era. We aimed to determine and compare the accessibility, content, and accreditation details of the various orthopedic fellowship programs available at the Match website and alternative online resources.

Methods: We studied eight subspecialties (Adult Reconstruction, Musculoskeletal Oncology, Foot and Ankle, Pediatric Orthopedics, Shoulder and Elbow, Orthopedic Spine Surgery, Sports Medicine, and Trauma) in a cross-sectional design during August/September 2019 for programs starting in July/August 2021. We registered the available baseline information at the SF Match site under various categories. We tried to reach the program-specific webpage through SF Match hyperlink and categorized our results into successful (direct and indirect) links and unsuccessful links with subcategorization. We also analyzed the information available at sponsoring society, FREIDA (Fellowship and Residency Electronic Interactive Database), and ACGME (Accreditation Council for Graduate Medical Education) websites.

Result: We analyzed 465 programs (874 positions) available through the SF Match website. A standardized program description was available for >80% of the programs in each subspecialty. The availability of a successful link for the program-specific webpage ranges from 35% (Pediatric Orthopedics) to 77% (Sports Medicine). Indirect links were almost twice as common as direct links. The success rates through the sponsoring society webpages vary from 3% (Shoulder and Elbow) to 53% (Pediatric Orthopedics). Failure rates after trying both (the Match and Society links) range from 10% (Musculoskeletal Oncology) to 34% (Shoulder and Elbow). FREIDA provides comprehensive information but is limited to accredited programs. ACGME accreditation rate varied from 14.6% (Foot and Ankle) to 98.9% (Sports Medicine).

Conclusion: The selection of a subspecialty fellowship program is crucial for most applicants. There are plenty of resources for the orthopedic fellowship programs' online presence after two decades since the first orthopedic fellowship match inception. Match website is the primary resource for the applicants. All parties could be benefited if both the programs and the sponsoring societies offer adequate online information to the Match, leading to ideal fellow-program matches and improved educational experiences. Our study may stand as a reference for future comparison possibly due to post-COVID evolution in the Match process. We recommend that consistent availability of direct functional program website links, point-based program description, and filter/comparison options may further improve online accessibility and quality of the content of the Match website.

## Introduction

Orthopedic surgery is considered among the highly competitive medical specialties to get in as a career in the United States [1]. Orthopedic surgery training consists of a five-year-minimum orthopedic surgery residency program, followed by optional subspecialty fellowship training. In the past, the real dilemma was whether to practice general orthopedics or subspecialty. However, in the last decade, post-orthopedic residency training in the form of subspecialty fellowship has become a norm rather than a trend [2]. SF Match (San Francisco Match) has been a trusted residency and fellowship match service for over 40 years in the United States. It currently conducts two residency matches and 22 specialty/sub-specialty matches throughout a calendar year including the orthopedic fellowship match [3]. The goal of the Match is to coordinate appointments, thus relieving the pressure of uncoordinated appointments and forced early choices. Centralized Application Service (CAS) makes the processing, distribution, and review of applications easy for the programs. The Match is designed to be a transparent, unbiased process in which applicants are matched to the fellowship program available at the highest level of their rank list on a competitive basis. The SF Match office neither sponsors nor approves or endorses any of the participating programs. The role of the Match is strictly limited to processing the match, while various sub-specialty societies are the programs' official sponsors (along with the available grant). The Accreditation Council for Graduate Medical Education (ACGME) is the non-profit body responsible for accrediting all graduate medical training programs (i.e., internships, residencies, and fellowships) for physicians in the United States since 1981 [4]. The Fellowship and Residency Electronic Interactive Database (FREIDA) is an online database provided at no charge by the American Medical Association (AMA) for ACGME-accredited graduate medical education programs [5].

In 2008, four orthopedic subspecialties started participating in SF Match for the first time. By 2016, a total of eight subspecialties were involved in the match as follows: (1) Adult Reconstruction (Hip and Knee Arthroplasty), (2) Musculoskeletal Oncology (MSK Onco.), (3) Foot and Ankle (F&A), (4) Pediatric Orthopedics (Peds Ortho.), (5) Shoulder and Elbow (S&E), (6) Orthopedic Spine Surgery, (7) Sports Medicine (Arthroscopy), and (8) Trauma. In 2018, application deadlines, interview deadlines, and match result days for all the orthopedic subspecialties were made uniform like National Residency Match Program (NRMP). There have been various studies in the past to determine the online accessibility and scarcity of content in orthopedic fellowship program-specific websites for individual subspecialty sponsor websites and discuss its impact on the fellow-program selection process [6-13]. However, we could not find any study primarily comparing various orthopedic subspecialties. This study aims to determine and compare the online accessibility, content, and accreditation details of the various orthopedic subspecialty fellowship programs available at the Match website and to review the alternate resources. In the era of virtual interaction due to the COVID pandemics where virtual program tours and introduction webinars are already being conducted, our study highlights an important subject and the scope of improvisation.

## Materials and methods

**Figure 1:**
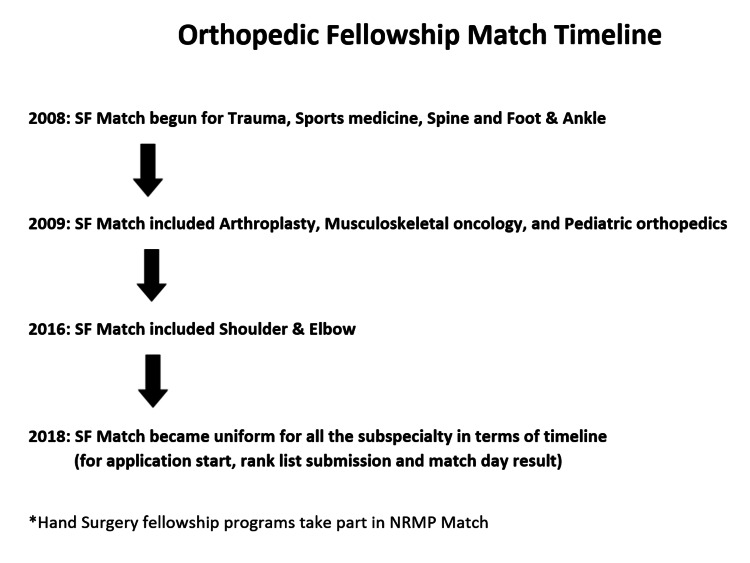
Timeline for inclusion of various subspecialties in Orthopedic Fellowship Match SF Match, San Francisco Match; NRMP, National Resident Matching Program.

We studied all eight orthopedic subspecialties available through the SF Match website (Figure 1 and Table 1).

**Table 1:** Details of various orthopedic subspecialty fellowship sponsoring societies *American Association of Hip and Knee Surgeons (AAHKS) is also sponsoring Musculoskeletal Oncology fellowships besides Adult Reconstruction as of 2021.

Orthopedic Subspecialty	Sponsoring Society (Full Name)	Short-Form	Foundation/Official Reformation Year
Adult Reconstruction	American Association of Hip and Knee Surgeons	AAHKS^*^	1991
Foot and Ankle	American Orthopedic Foot and Ankle Society	AOFAS	2001
Musculoskeletal (MSK) Oncology	Musculoskeletal Tumor Society	MSTS	1977
Pediatric Orthopedics	Pediatric Orthopedic Society of North America	POSNA	1984
Shoulder & Elbow	American Shoulder and Elbow Surgeons	ASES	1984
Spine Surgery	North American Spine Society	NASS	1985
Sports Medicine	Arthroscopy Association of North America	AANA	1978
	American Orthopedic Society for Sports Medicine	AOSSM	1972
Trauma	Orthopedic Trauma Association	OTA	1985

Adult Reconstruction (Hip and Knee) and MSK Oncology were considered as a single subspecialty in the SF Match. However, we have studied them separately as they have different affiliated societies and involve the treatment of different pathologies. We did not include the orthopedic hand surgery fellowship program as it participates in the NRMP instead of the SF Match, and it is open for general surgery and plastic surgery residents along with orthopedic residents.

We explored the Match website (www.sfmatch.org) from August 5, 2019, to September 4, 2019, for the Orthopedic Fellowship Programs starting in July/August 2021. The period selected for online data collection was a typical application window for the upcoming match cycle. We used the Google Chrome® search engine (v. 76.0.3809), and we registered the baseline information of the SF Match website, which is uniform for all the fellowship programs irrespective of subspecialty. Afterward, we went through various subspecialties to collect data under different information headings. Besides primary program information (basic information, contact, personnel details, and timelines), we extended our search for program descriptions and functionality of available program-specific website links. We reviewed the Match program description for each program to get a subjective idea about the program. We also analyzed relevant information like the percentage of programs where application deadline/interview dates were missing and whether research opportunity was available besides clinical training.

**Figure 2:**
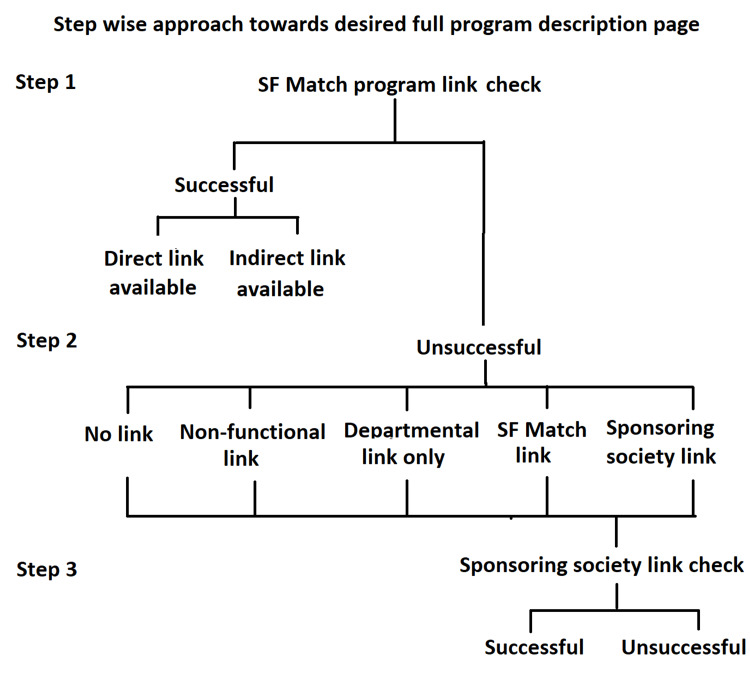
Flowchart detailing of the sequential three-step approach for online accessibility of desired program-specific page through SF Match and sponsoring society webpages Definitions: Departmental link only - no extension toward the program webpage/description; SF Match link - link back to the SF Match; sponsoring society link - sponsoring society’s main page that required further search. SF Match, San Francisco Match.

The desired webpage was defined as the program-specific web page where the host institute provided a full program description. We used a sequential three-step approach to look for the desired page (Figure 2).

Whenever the functional link was found on the SF Match web page, we registered whether it is a direct or indirect link. The indirect link was defined as a requirement of more than one click from the Match webpage to reach the desired page through the institutional website. An unsuccessful attempt to reach the desired page was categorized as one of the following five: (1) no link, (2) non-functional link (e.g., web page not found), (3) departmental link only (without any extension toward full program description), (4) link back to the SF Match (SF Match link/webpage), and (5) link to the sponsoring society (sponsoring society web page that required further search). Another reviewer also checked the unsuccessful departmental links before declaring them as a departmental link only without extension toward the desired web page. Irrespective of the category of such an unsuccessful attempt, we extended our search via the sponsoring society webpage and tried to reach up to the desired page. We recorded whether the sponsoring society webpage helps to reach up to the desired page if the attempt was failed or was unsuccessful through the Match website. We did not perform any random Google search with program names for the unsuccessful category or did not analyze program-specific web page contents.

Besides the Match, we also explored alternative resources available online. These included the FREIDA website (freida.ama-assn.org), ACGME website (apps.acgme.org), and sponsoring society website. We again registered uniform baseline information from these resources. We compared the current ACGME accreditation rate of programs in various subspecialties with the results published in 2014 by Daniels et al. [14]. We also analyzed the withdrawal of ACGME accreditation.

## Results

In total, 465 fellowship programs offering 874 fellowship positions were identified (Table 2).

**Table 2:** Details of various orthopedic subspecialty program numbers, positions, and their listing in FREIDA, ACGME, and society websites FRIEDA, Fellowship and Residency Electronic Interactive Database; ACGME, Accreditation Council for Graduate Medical Education; AAHKS, American Association of Hip and Knee Surgeons; AOFAS, American Orthopedic Foot and Ankle Society; MSTS, Musculoskeletal Tumor Society; POSNA, Pediatric Orthopedic Society of North America; ASES, American Shoulder and Elbow Surgeons; NASS, North American Spine Society; AANA, Arthroscopy Association of North America; AOSSM, American Orthopedic Society for Sports Medicine; OTA, Orthopedic Trauma Association.

Orthopedic Subspecialty	No of Programs (n)	No of Positions	Mean Positions per Program	Programs Listed in FREIDA Webpage	Programs Listed in ACGME Webpage	Sponsoring Society	Programs Listed in Society Webpage
Adult Reconstruction	100	199	1.99	24 (24%)	37 (37%)	AAHKS	100 (100%)
Foot and Ankle	48	77	1.60	07 (14.6%)	12 (25%)	AOFAS	48 (100%)
MSK Oncology	20	26	1.30	12 (60%)	14 (70%)	MSTS	20 (100%)
Pediatric Orthopedics	45	73	1.62	25 (55.5%)	40 (88.9%)	POSNA	44 (97.8%)
Shoulder & Elbow	29	39	1.34	0 (0%)	00 (0%)	ASES	29 (100%)
Spine Surgery	71	135	1.90	17 (24%)	27 (38%)	NASS	54 (76%)
Sports Medicine	91	234	2.57	90 (98.9%)	129^*^(141%)	AANA/AOSSM	87 (95.6%)/83 (91.2%)
Trauma	61	91	1.49	11 (18%)	16 (17.5%)	OTA	50 (82%)

The mean number of fellowship positions per program was highest in Orthopedic Sports Medicine (2.57) and lowest in MSK Oncology (1.30). FREIDA website listed only current ACGME-accredited programs; however, the ACGME website listed programs with current or past ACGME accreditation. For Sports Medicine, the ACGME web page showed 30 more programs that were not listed in the Match, and this discrepancy may be due to the termination of the previously existing programs. Among sponsoring society websites, North American Spine Society (NASS), Sports Medicine Societies (American Orthopaedic Society for Sports Medicine [AOSSM] and Arthroscopy Association of North America [AANA]) and Orthopaedic Trauma Association (OTA) were having some missing programs. All other subspecialty society websites listed all the programs available for the match (except one missing program). Thus, sponsoring society’s website proved to be the second-best option after the Match web page for full program description search.

The Match website provided a standardized program description window for each program (Table 3).

**Table 3:** Comparison of availability of uniform program information at SF Match webpage for various orthopedic subspecialties SF Match, San Francisco Match; MSK, musculoskeletal.

Orthopedic Subspecialty	Program Description	Research Opportunity	Application Deadline	Interview Dates	Coordinator, Program Director, and Chair	Address, Phone, Fax, and Email	Training Positions, Duration, and Sequence
Adult Reconstruction (n = 100)	81 (81.0%)	56 (56.0%)	82 (82.0%)	64 (64.0%)	Available for all the programs in each subspecialty (100%)		
Foot and Ankle (n = 48)	48 (100%)	21 (43.7%)	44 (91.6%)	39 (81.2%)			
MSK Oncology (n = 20)	17 (85.0%)	05 (25.0%)	16 (80.0%)	11 (55.0%)			
Pediatric Orthopedics (n = 45)	41 (91.1%)	13 (28.9%)	38 (75.5%)	36 (80.0%)			
Shoulder and Elbow (n = 29)	24 (82.7%)	24 (82.7%)	25 (68.9%)	25 (68.9%)			
Spine Surgery (n = 71)	64 (90.1%)	61 (95.3%)	62 (87.3%)	44 (61.9%)			
Sports Medicine (n = 91)	75 (82.4%)	18 (24.0%)	79 (86.8%)	79 (86.8%)			
Trauma (n = 61)	66 (92.4%)	31 (46.9%)	55 (90.1%)	35 (57.3%)			

It always consisted of basic program details (name, ID, number of positions, duration, and type of training sequence), contact details (address, phone, fax, and email), personnel information (coordinator, program director, and chair), and timelines (application deadline and interview dates). The program description was available for most of the programs (>80%) in each subspecialty. The program descriptions were highly variable. It usually had information regarding the fellowship curriculum, current ACGME accreditation status, and eligibility criteria. The program description available on the Match webpage ranges from 81% (Adult Reconstruction) to 100% (Foot and Ankle). The availability of described research opportunities (besides clinical training) ranges from 25% (MSK Oncology) to 95.3% (Spine Surgery).

The results of a three-step sequential search for the desired program-specific webpage for each subspecialty are shown in Table 4.

**Table 4:** Results of sequential three-step approach to reach up to the desired program-specific webpages via SF Match and sponsoring society websites SF Match, San Francisco Match.

Orthopedic Subspecialty	Link Toward the Desired Page Via SF Match Webpage									Success (in an Unsuccessful Category) Via Society Webpage	Failure in Finding the Desired Page
	Successful			Unsuccessful							
	Direct Link	Indirect Link	Total Successful Links	No Link	Non-functional Link	Dept. Link Only	SF Match Link	Sponsor Society Link	Total Unsuccessful Link		
Adult Reconstruction (n = 100)	20	42	62 (62%)	09	12	14	03	0	38 (38%)	14 (14%)	24 (24%)
Foot and Ankle (n = 48)	11	23	34 (70%)	05	02	06	01	0	14 (30%)	03 (6%)	11 (23%)
MSK Oncology (n = 20)	03	10	13 (65%)	02	04	00	01	0	07 (35%)	05 (25%)	02 (10%)
Pediatric Orthopedics (n = 45)	09	07	16 (35%)	03	04	00	22	0	29 (65%)	24 (53%)	05 (11%)
Shoulder and Elbow (n = 29)	05	13	18 (62%)	08	01	02	00	0	11 (38%)	01 (3%)	10 (34%)
Spine Surgery (n = 71)	16	29	45 (63%)	11	09	06	00	0	26 (37%)	03 (4%)	23 (32%)
Sports Medicine (n = 91)	16	54	70 (77%)	05	06	01	09	0	21 (23%)	05 (5%)	16 (17%)
Trauma (n = 61)	08	34	42 (68%)	04	04	03	01	7	19 (32%)	05 (8%)	14 (22%)

The availability of a successful link for the desired page at the Match webpage ranges from 35% (Pediatric Orthopedics) to 77% (Sports Medicine). For most subspecialties, the availability of successful indirect links was almost twice as common as the direct links except for Pediatric Orthopedics for which it was almost equal. The success rate of sponsoring society webpage to reach out for the desired webpage in the unsuccessful category was quite variable, ranging from 3% (Shoulder and Elbow) to 53% (Pediatric Orthopedics) among the various subspecialties. The failure rate of reaching the desired webpage despite trying both (the Match and the Society) websites ranges from 10% (MSK Oncology) to 34% (Shoulder and Elbow).

FREIDA website has the facility to filter programs by geographic region (or state), program type (university, hospital or military-based, etc.), qualification (US MD, DO, IMG), visa sponsorship (H1B/J-1), salary, and working hours to narrow the search. FREIDA was uniform to provide three types of information. (1) Overview includes program location, positions, timelines, website, etc. (2) Faculty and fellow information includes exam/score requirements (USMLE/COMLEX), faculties (fulltime/part-time and physician/non-physician), fellows (US MD/DO/IMG, male/female) and work and call schedules. (3) Program environment includes educational benefits (research, management, finance, rotation, elective, teaching, etc.), insurance benefits (medical, dental, life, disability, etc.), time-off (vacation, sick days, etc.), employment benefits (allowances for housing, moving, parking, childcare, conferences, technology, etc.), and evaluation information (for fellow and program). The ACGME accreditation analysis of various subspecialties is shown in Table 5.

**Table 5:** ACGME accreditation details and comparison between various orthopedic fellowship subspecialties *Program number (higher than the total available for the match) may include terminated programs along with the active programs. ACGME, Accreditation Council for Graduate Medical Education; MSK, musculoskeletal.

Orthopedic Subspecialty	ACGME-Accredited Programs for 2021	ACGME Non-accredited Programs for 2021	Programs Listed With ACGME Website	Withdrawal of ACGME Accreditation			ACGME-Accredited Program % in 2014^*^
				Voluntary	Disciplinary	Total	
Adult Reconstruction (n = 100)	24 (24%)	76 (76%)	37 (37%)	12	1	13 (13%)	39.6%
Foot and Ankle (n = 48)	07 (14.6%)	41 (85.4%)	12 (25%)	05	0	05 (10.4%)	16.3%
MSK Oncology (n = 20)	12 (60%)	08 (40%)	14 (70%)	01	1	02 (16.6%)	78.6%
Pediatric Ortho. (n = 45)	25 (55.5%)	20 (44.5%)	40 (88.9%)	14	1	15 (33%)	59.5%
Shoulder and Elbow (n = 29)	00 (0%)	29 (100%)	-	-	-	-	-
Spine Surgery (n = 71)	16 (22.5%)	55 (77.5%)	27 (38%)	09	2	11 (40.7%)	30%
Sports Medicine (n = 91)	90 (98.9%)	01 (1.1%)	129^*^(141%)	37	2	39 (30.2%)	93.1%
Trauma (n = 61)	11 (18%)	50 (82%)	16 (17.5%)	05	0	05 (8.1%)	17.9%

The highest percentage of fellowship programs accredited by the ACGME was in Orthopedic Sports Medicine (98.9%), compared to the lowest (14.6%) in Foot and Ankle. There are very handful of ACGME-accredited programs available for reconstruction/sports for the upper extremity. All Shoulder and Elbow subspecialty programs listed for the match are non-accredited. The ACGME accreditation rate of seven out of eight subspecialties has decreased (up to 18.6%) in the last five years (2014-2019) when compared with a study published in 2014 [14]. However, the Sports Medicine fellowship accreditation rate has increased from 93.1% to 98.9%. Highlights of program-specific webpage information in each society webpage are described in Table 6.

**Table 6:** Highlight of fellowship program information available at the sponsoring society webpage for different orthopedic subspecialties AAHKS, American Association of Hip and Knee Surgeons; AOFAS, American Orthopedic Foot and Ankle Society; MSTS, Musculoskeletal Tumor Society; POSNA, Pediatric Orthopedic Society of North America; ASES, American Shoulder and Elbow Surgeons; NASS, North American Spine Society; AANA, Arthroscopy Association of North America; AOSSM, American Orthopedic Society for Sports Medicine; OTA, Orthopedic Trauma Association; MSK, musculoskeletal; IMG, international medical graduate; ER, emergency room; ECFMG, Educational Commission for Foreign Medical Graduates.

Orthopedic Subspecialty	Sponsoring Society	Highlights of Program Information Available at Society Webpage
Adult Reconstruction	AAHKS	Basic program details along with website link
Foot and Ankle	AOFAS	Detailed program description of profile and curriculum with information for IMG (non-US/Canada) acceptance
MSK Oncology	MSTS	Interview dates and website link only
Pediatric Orthopedics	POSNA	Basic program details, website link, and information of IMG (ECFMG-certified) acceptance
Shoulder and Elbow	ASES	Program location and director/coordinator/faculty names with a website link
Spine Surgery	NASS	Detail program description with goals and precise information in terms of work frame (clinical, non-clinical, research), region (cervical, thoracic, lumbar), and etiology (degenerative, trauma, pediatric, tumor)
Sports Medicine	AANA	Program name and location (state) only
	AOSSM	Basic program details with the website link
Trauma	OTA	Detailed program description with Trauma center details (Level and No. of beds, yearly admission/ER visits/procedures) and case-load description (common fractures, arthroplasty, external fixator, debridement, non-union repair, etc.)

## Discussion

The selection of a subspecialty fellowship program is crucial for most applicants. Several factors play a vital role in the selection such as the applicant’s personal interest, geographic location, financial impact, and the program itself [15]. Easy online accessibility, content, and accreditation status of programs can influence objective parameters for candidates to investigate the program, thus potentially affecting their decision to apply and compose their rank list [16-19]. The selective application process may help decrease interview costs for the applicant as well as for the program [20-24]. Reputed programs (in terms of region, number of positions, accreditation, and affiliation with top medical school/residency program/hospital) may or may not be affected by online information availability. However, the majority of new or remotely located programs are likely to be negatively impacted by the weak web presence [7,11,25]. The SF Match provides detailed program descriptions, and other resources are also available for candidates to review.

FREIDA provides comprehensive information regarding programs but is only limited to ACGME-accredited programs. ACGME accreditation does standardize the programs for a minimum requirement and is considered one of the important factors in deciding the program selection. However, many orthopedic fellowship programs that are not ACGME accredited are available in the match. ACGME website is the most reliable source to confirm ACGME accreditation status because it provides up-to-date information, accreditation history, and withdrawal details if any. Except for Sports Medicine, the ACGME accreditation rate is not more than 60% for most subspecialties. The Shoulder and Elbow subspecialty is still not widely recognized as a subspecialty by ACGME. Moreover, there have been many programs for which ACGME accreditation has been withdrawn in the last few years. Spine Surgery, Sports Medicine, and Pediatric Orthopedics were among the top three subspecialties with decreasing overall ACGME accreditation rate when compared to a study by Daniels et al. in 2014 [14]. Most of the time, the withdrawals have been voluntary rather than disciplinary. The accreditation rate of all the subspecialties (except Sports Medicine) has decreased in the last five years (2014-2019) with the highest reduction (18.6%) in Adult Reconstruction. ACGME accreditation may help define uniform standards for fellowship training, but it is unproven that it is always preferred over the other. The possible disadvantages of ACGME accreditation include duty-hour restrictions, intensive documentation and paperwork requirements, comprehensive facility and faculty requirements, and insurer regulations limiting the billing for ACGME-accredited fellows [14]. On the other hand, some non-accredited programs may offer different exposure, high volume, and more independence with staff privileges [14].

After the Match, the sponsoring society’s website seems to be the second-best place to find the program information. However, a detailed program description was available only for half of the subspecialties at sponsoring society websites: (1) the American Orthopaedic Foot & Ankle Society (AOFAS), (2) Pediatric Orthopaedic Society of North America (POSNA), (3) North American Spine Society​​​​​​​ (NASS), and (4) Orthopaedic Trauma Association​​​​​​​ (OTA). The remaining four sponsoring society websites contain very limited or basic information. OTA has covered the trauma program description in a precise and meticulous way. However, international medical graduate (IMG) acceptance details were missing, which are covered by AOFAS and POSNA. Moreover, OTA covers 82% of programs, and NASS includes only 76% of programs of all participating in the Match. Thus, there is no option left but to look for the institutional website's program description for the remaining programs. SF Match webpage provides a successful link for 35%-77% of various orthopedic subspecialties. Again, almost two-third of successful links were indirect links. We found that search for the following terms was helpful to reach the desired page via the indirect link: ‘Education’, ‘Graduate Medical Education (GME)’, ‘Fellowship’, ‘Orthopedic Fellowship’, ‘Career’, ‘Residency training’, and ‘Other’. Among the unsuccessful/broken links, most of the time, there was no link or non-functional link. Links reaching up to department webpage can be time-consuming without end-result if proper search terms are not used. Rarely, there is just the SF Match homepage link (Pediatric Orthopedics and Sports Medicine) or the sponsoring society homepage link (Trauma) available instead of the program-specific webpage link. Overall, a failure rate of 10%-34% to reach out to the desired program webpage indicates the scope for improvisation.

Besides, as per our knowledge, there are more than 10 orthopedic fellowship programs available across the United States, which are not participating in the match mainly because of the atypical nature of the subspecialty including but not limited to Hip Preservation, Limb Deformity Correction, Metabolic Bone Disease, and Geriatric Trauma. For such programs, the program-specific institutional webpage is mostly the only available option to get the program information.

There were some limitations to this study. The accessibility evaluation was based on the SF Match, FREIDA, ACGME, and sponsoring society websites. We did not explore ABOS, AAOS, and direct search for program-specific webpages to limit our dataset. The online evaluations were performed during a fixed period. There is a chance of human or server error during website content review during the study. The Match program description section was not comparable because of discrete information instead of point-based attributes.

## Conclusions

To conclude, there are plenty of resources for the orthopedic fellowship programs' online presence after two decades since the first orthopedic fellowship match inception. Both parties (applicant and the program) could be benefited from adequate online information, leading to ideal fellow-program matches and improved educational experiences. Considering the organizational limitation of FREIDA, ACGME, and sponsoring societies, we believe that the Match website is the primary resource for the applicants. However, further coordination between participating programs and the sponsoring organizations may still improve online accessibility. Moreover, keeping the information up to date is also equally important. While this study pertained to orthopedic fellowships, the information obtained may be relevant for subspecialties outside of orthopedics. Our study may also stand as a reference for future comparison, possibly due to post-COVID evolution in the Match process. We recommend the following suggestions for the Match website that may further improve the online accessibility and quality of content: (1) Consistent availability of direct and a functional program website link as there is no alternative to it for complete authentic information; (2) point-based program description for important information; (3) addition of filters for important program attributes (e.g., ACGME accreditation) besides currently available filters of state and application deadline; (4) provision of fellowship program comparison option within the subspecialty; and (5) direct video link for the program introduction if made available by the program.
